# Ki-67 assessment in early breast cancer: SAKK28/12 validation study on the IBCSG VIII and IBCSG IX cohort

**DOI:** 10.1038/s41598-019-49638-4

**Published:** 2019-09-19

**Authors:** Zsuzsanna Varga, Qiyu Li, Wolfram Jochum, Ulrike Perriard, Tilman Rau, Jean-Christoph Tille, Hanne Hawle, Dirk Klingbiel, Beat Thuerlimann, Thomas Ruhstaller

**Affiliations:** 1 Department of Pathology and Molecular Pathology, University Hospital Zurich, Zurich, Switzerland; 2 SAKK Coordinating Center, Bern, Switzerland; 3 Institute of Pathology, Cantonal Hospital, Gt. Gallen, Switzerland; 4 The Cantonal Institute of Pathology, Locarno, Switzerland; 5 Institute of Pathology, University Hospital Bern, Bern, Switzerland; 6 Division of Clinical Pathology, University Hospital Geneva, Geneva, Switzerland; 7 Breast Center, St. Gallen, Cantonal Hospital, St. Gallen, Switzerland

**Keywords:** Predictive markers, Surgical oncology

## Abstract

The assessment of Ki-67 in early-stage breast cancer has become an important diagnostic tool in planning adjuvant therapy, particularly for the administration of additional chemotherapy to hormone-responsive patients. An accurate determination of the Ki-67 index is of the utmost importance; however, the reproducibility is currently unsatisfactory. In this study, we addressed the predictive/prognostic value of Ki-67 index assessed by using the most reproducible methods, which were identified in the pilot phase. Paraffin blocks obtained from patients with moderately differentiated, estrogen receptor (ER)-positive early-stage breast cancer in Switzerland, who were originally randomized to the treatment arms with and without chemotherapy in the IBCSG VIII-IX trials, were retrieved. Of these 344 randomized patients, we identified 158 patients (82 treated with and 76 treated without chemotherapy) for whom sufficient tumour tissue was available. The presence of Ki-67 was assessed visually by counting 2000 cells at the periphery (A) and estimating the number of positive cells in five different peripheral regions (C), which was determined to be the most reproducible method identified the pilot phase. The prognostic and predictive value was assessed by calculating the breast cancer-free interval (BCFI) and overall survival (OS) rate. Ki-67 was considered a numerical and categorical variable when different cut-off values were used (10%, 14%, 20% and 30%). An mRNA-based subtyping by using the MammaTyper kit with the application of a 20% Ki-67 immunohistochemistry (IHC) cut-off equivalent was also performed. 158 of 344 randomized patients could be included in the Ki-67 analysis. The mean Ki-67 values obtained by using the two methods differed (A: 21.32% and C: 16.07%). Ki-67 assessed by using method A with a cut-off of 10% was a predictive marker for OS, as the hazard ratio (>10% vs. <=10%) in patients with chemotherapy was 0.48 with a 95% confidence interval of [0.19–1.19]. Further, the HR of patients treated without chemotherapy was 3.72 with a 95% confidence interval of [1.16–11.96] (p_interaction_=0.007). Higher Ki-67 index was not associated with outcome and using the 10% Ki-67 cut-off there was an opposite association for patients with and without chemotherapy. Ki-67 assessments with IHC significantly correlated with MammaTyper results (p=0.002). The exact counting method (A) performed via a light-microscope revealed the predictive value of Ki-67 assessment with a 10% cut-off value. Further analyses employing image analyses and/or mRNA-based-assessments in larger populations are warranted.

## Introduction

The assessment of proliferation by estimating the Ki-67 labelling index has increasingly become an integral biomarker of early-stage breast cancer^1–13^. The decision on further adjuvant hormonal therapy with additional chemotherapy in *luminal A-* and *B-like* breast cancers is based on the progesterone receptor status and Ki-67 labelling index^1–3,7,10,12–15^. Recent indications for preoperative chemotherapy, including patients with luminal-type breast cancers, increasingly include the Ki-67 labelling index as a biomarker for this therapy choice^9,16,17^. The issues of reproducibility and the choice of the best method for measuring the Ki-67 labelling index have been the subject of several pathology studies and were included in several oncological/senological guidelines^4,5,8,11,18–28^. Difficulties in reproducing the Ki-67 labelling index are particularly crucial in the intermediate range proliferative *luminal B-like* breast cancers, as published data are reaching a consensus on this delicate issue and advising caution with the use of this biomarker^4,5,8,11,18–20,22–28^. Data have accumulated and results supporting or refuting the superiority of a digital or visual analysis are approximately equal, basically suggesting that both methodologies can be applied for routine diagnostic purposes^3,22,23,27–29^.

We previously conducted a reproducibility study (SAKK 28/12 pilot phase) testing different Ki-67 methods using visual and digital analyses to identify the most reproducible method regarding intra- and inter-rater reliability. In the SAKK 28/12 validation phase, the chosen methods from the pilot phase were subjected to further analysis using a prospective clinical cohort comprised of paraffin blocks obtained from patients who were initially enrolled in the IBCSG VIII and IX trials^11,13,22–28,30,31^.

The aim of this study was to correlate the immunohistochemical Ki-67 labelling index obtained using the two most reproducible methods from the SAKK 28/12 study with clinical data such as overall survival (OS) and the breast cancer-free interval (BCFI)^27^. Additionally, we determined the Ki-67 index with an mRNA-based assessment using MammaTyper and correlated the mRNA levels with OS and BCFI. The reason to include mRNA-based subtyping and Ki-67 mRNA values was the high interobserver reliability and interclass correlation reported previously in mRNA-based subtyping^32^.

## Methods

### Objectives of the study

The main goal of the validation phase of SAKK 28/12 is to determine the prognostic/predictive value of Ki-67, which was assessed by using the most reproducible methods, as identified in the pilot phase (methods A and C), for predicting OS and BCFI^27^. In the pilot phase, two assessing methods resulted in an almost equally high inter-observer reliability, which were both chosen for further validation in this study. These methods were: A (exact counting as the original recommendation) and C (estimating resp. eyeballing in central and peripheral regions)^27^.

Additionally, we aim to assess the association between mRNA-based subtyping and assessments based on methods A/C. Furthermore, we are also interested in determining the associations between the Ki-67 mRNA level and OS/BCFI.

## Materials and Methods

We retrieved residual paraffin blocks collected before study treatment from patients who were enrolled in the IBCSG VIII and IX studies and registered in Switzerland.

The designs of IBCSG Trials VIII and IX have been described in detail elsewhere^33,34^. IBCSG Trials VIII and IX were randomized clinical trials that compared the effectiveness of adjuvant endocrine therapy alone and sequential chemotherapy followed by endocrine therapy for node-negative invasive breast cancer among pre- and peri-menopausal (Trial VIII) and post-menopausal (Trial IX) women^33,34^. The breast cancer-free interval was defined as the length of time from the date of randomization to any invasive breast cancer relapse (including ipsilateral or contralateral breast recurrence) or was censored at date of the last follow-up or death without relapse. OS was defined as the length of time from the date of randomization to death from any cause or censored at the last known date the patient was alive^33,34^.

Briefly, from 1990–1999, in Trial VIII, 1063 pre- and peri-menopausal women with node-negative early breast cancer were randomly assigned to endocrine therapy with 24 months of goserelin alone, six cycles of chemotherapy with classical cyclophosphamide, methotrexate and 5-fluorouracil (CMF), or a sequence of 6 cycles of CMF followed by 18 months of goserelin. Similarly, from 1988–1999, in Trial IX, 1669 eligible post-menopausal women were randomly assigned to endocrine therapy with 5 years of 20 mg of tamoxifen daily or 3 cycles of CMF followed by tamoxifen to complete 5 years therapy. In each trial, randomization was stratified according to the locally determined ER status. Patient follow-up, vital status and the date of any relapse or recurrence are recorded in the IBCSG database. The median follow-up from randomization in Trial VIII is 12 years and in Trial IX 13 years^33,34^. Ethical approval was obtained in participating countries according to national regulations.

**Figure 1:**
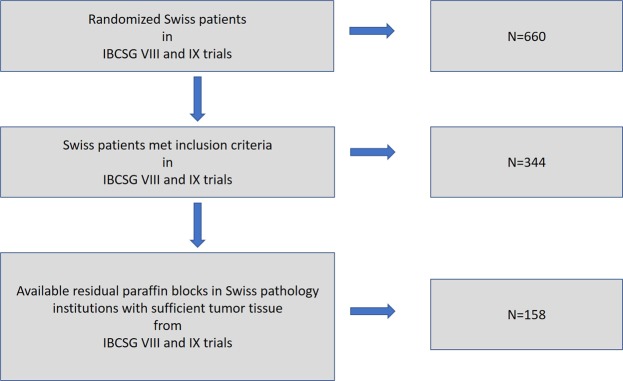
Flow-chart diagram of patients and sample selection from IBCSG VIII and IX trials.

Originally, 660 patients in Switzerland, all with G2 tumors, were randomized in the IBCSG VIII and IX studies, and 344 of these patients met the inclusion criteria (as ER positive, G2). Clinical outcome data was available only for the patients who met the inclusion criteria in the study (Fig. 1). Paraffin blocks from 158 of 344 Swiss patients were retrieved from the archives of Swiss pathology institutions and contained sufficient amounts of invasive breast cancer tissues; 82 of these patients were randomized to the treatment arm with chemotherapy. Eight pathology institutions (University Hospital Lausanne, University Hospital Basel, University Hospital Bern, University Hospital Geneva, University Hospital Zurich, Cantonal Hospital St. Gallen, Cantonal Hospital Graubünden and Cantonal Hospital Locarno Switzerland) that originally participated the IBCSG VIII and IX studies provided paraffin blocks. Patients selected for this study had a moderately differentiated hormone receptor-positive breast cancer (with a negative Her2 status available in the original studies). Morphology was controlled by preparing a fresh haematoxylin-eosin (HE)-stained section to confirm the presence of invasive cancer available for further studies. Data on overall survival (OS) and the breast cancer-free interval (BCFI) were provided to the SAKK by the IBCSG.

This project is a part of a retrospective breast cancer study on archived human tissues and was approved by the Ethical Committee of the Canton Zurich (ZH-KEK-2012-553).

### Immunohistochemistry for Ki-67

The Ki-67 status was analysed using immunohistochemical staining, as described previously^27^. Briefly, sections for Ki-67 were stained centrally in the Institute of Pathology and Molecular Pathology, University Hospital Zurich Switzerland according to the following laboratory protocol (the protocol is from the Institute of Pathology and Molecular Pathology, University Hospital Zurich, Switzerland, Laboratory for *in situ* technology): Two micrometer thick sections were freshly cut from paraffin blocks containing a sufficient amount of invasive carcinoma tissue. Ki-67 staining was performed using the fully automated Benchmark staining system (Ventana Medical Systems) and the primary antibody (rabbit monoclonal anti-Ki-67 human, clone 30-09 Ventana Medical Systems, Inc.).

### Interpretation of Ki-67 Immunohistochemistry

As discussed in the pilot phase of the study, the most reproducible methods were applied to this cohort and were conducted by the principle investigator (ZV) of this study, who was blinded to the clinical outcome (OS/BCFI) and performed the evaluations via a light microscope^27^. The assessment methods designated as the best methods were method A (exact counting) and method C (eyeballing). Both methods were scored by the principle investigator (ZV).

Method A was defined as the original method of counting 2000 invasive cells in randomly selected, high-power magnification (400×) fields at the periphery of the tumor and determining the percentage of Ki-67 staining^12^.

Method C was defined as an estimating (so-called eye-balling) assessment analysis performed via a light microscope at 20× magnification for five random fields within the tumor (both the periphery and center), which included approximately 500 cells.

Digital analysis was not applied in this study, as none of the digital analysis methods investigated in the pilot phase outperformed the best light microscopic methods, methods A and C, in terms of reproducibility.

Ki-67 values are reported as percentages of the invasive tumour cells. Throughout this paper, the percent symbol will be removed for Ki-67 index to simplify the presentation. Therefore, the Ki-67 index measured by using methods A and C are presented as a number ranging from 0 to 100.

### Assessment of the Ki-67 mRNA

All 158 paraffin blocks underwent an assessment of the Ki-67 mRNA using the MammaTyper assay, as described previously^32^. Briefly, ten micrometer thick, unstained slides were freshly cut from the paraffin blocks at the Institute of Pathology and Molecular Pathology of the University Hospital Zurich and were sent to BioNTech Diagnostics GmbH, Mainz, Germany for the MammaTyper analyses. The mRNA was extracted from the unstained slides with the RNXtract RNA Extraction Kit (BioNTech Diagnostics) and was subsequently measured via the MammaTyper analysis using the same technical procedures described in a previous study^32^. The mRNA analysis was blinded to the values of the Ki-67 immunostaining and the clinical outcome. The results were obtained from 137 paraffin blocks for the study. The remaining 21 blocks were excluded either due to a low RNA content because of a poor tissue quality or to missing clinicopathological information.

### Interpretation of the Ki-67 mRNA assessment

As described above, MammaTyper is a molecular *in vitro* diagnostic test for the quantitative detection of the mRNA expression of the *ERBB2* (HER2), *ESR1* (ER), *PGR* (PR) and *MKI67* (marker of proliferation Ki-67) genes. The test is used for the molecular subtyping of breast cancer tissue into the intrinsic subtypes *Luminal A-like*, *Luminal B-like* (HER 2-positive or -negative), *HER2-positive* (non-luminal) and *Triple negative* (ductal), as defined in the St. Gallen Consensus Conference recommendations utilized since 2011^1,7,14^. The immunohistochemistry cut-off for the differentiation between *Luminal A-like* and *Luminal B-like* cancers was prospectively set to 20%, and the MammaTyper kit was designed using this cut-off based on the mRNA values^32^.

### Statistical analysis

To assess the prognostic value of Ki-67 regarding to time-to-event endpoints (OS and BCFI), a Cox regression model with Ki-67 as the only covariable was fitted. To assess the predictive value of Ki-67, a Cox regression model including the Ki-67 index, treatment and the interaction of these two parameters was fitted. P values were calculated for these models by using Wald’s test. For additional research objectives, the Wilcoxon test was used to identify the association between Ki-67 index assessed by using methods A/C and the Ki-67 mRNA, while the log-rank test was used to assess the associations between the Ki-67 mRNA and OS/BCFI. The sample size estimation based on the prognostic value of Ki-67 (assessed by using method A) for BCFI was performed before we received the clinical data from IBCSG and retrieved the residual paraffin blocks. Assuming a rate of BC recurrence of 20%, a Cox regression analysis of Ki-67 with a standard deviation of 7.5 (estimated from the pilot phase of this project) based on a sample of 231 observations achieves 80% power at a 0.05 significance level to detect a hazard ratio of 1.25 and the number of observations accordingly.

PASS 11 was used to calculate the sample size. SAS 9.4 and R 3.3.2 were used for the analyses. Multiple test corrections were not applied to all p values; thus, the results are considered exploratory. The motivation to select the specific Ki-67cut-offs as 10,14,20,30 was based on previously published consensus recommendations^1–3,7,10,12–15^.

### Novelty and Impact statement

Our data draws attention to the fact that Ki-67 cut-off values are methodology and observer dependent, and median Ki-67 values can vary depending on the assessment methods. In our study, the exact counting under a light microscope revealed the predictive relevance of Ki-67 assessment using a 10% cut-off value for predicting OS or BCFI.

### Ethical approval and consent to participate

Ethical approval and informed consent from all patients to use the paraffin blocks at the time of IBCSG VIII and IX randomization were obtained according to the national regulations. The retrospective study was approved by the Lead Ethical Committee of the Canton Zurich (ZH-KEK-2012-553). All procedures performed in this study were conducted in accordance with the ethical standards of the institutional and national research committees and with the 1964 Declaration of Helsinki and its later amendments or comparable ethical standards.

### Consent for publication

All authors, as well the SAKK and the IBCSG scientific committees, read and approved the manuscript prior to submission. The study, including the design and data interpretation, was discussed during the SAKK annual and semiannual meetings.

## Results

### Summary

158 of 344 Swiss patients randomized in the IBCSG BIG VIII and IX trials with G2 hormone receptor-positive and Her2-negative breast cancer and with available tumor tissue in paraffin blocks were included in this study (Fig. 1). Our results show, as described in details below, that different Ki-67 assessment methodologies have different mean and median values and the methodologies influence the correlation between OS/BCFI and Ki-67 labelling index. We could demonstrate, that a cut-off of 10% using visual Ki-67 IHC assessment was a predictive marker of OS in patients who were not treated with chemotherapy. Moreover, we found that Ki-67 IHC assessments significantly correlated with Ki-67 mRNA measurements.

### Descriptive analysis of Ki-67 immunohistochemistry and clinical outcomes

#### Mean and median Ki-67 values obtained using immunohistochemical Methods A and C

**Figure 2:**
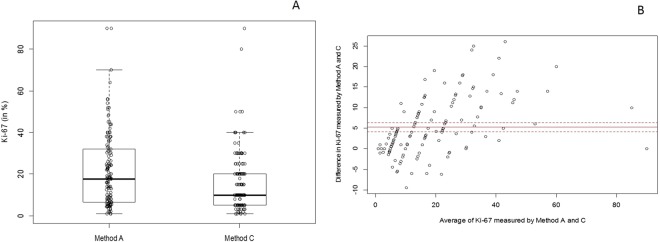
(**A)** Boxplots of Ki-67 levels assessed by using Methods A and C. (**B)** Bland-Altman plot of Methods A and C.

Compared with Method C (mean=16.07 and median=10.00), Method A (mean=21.32 and median=17.70) generally produces a higher Ki-67 value. Range for Ki-67 values in both Method A and C was 1.00 to 90.00. These differences are shown in Fig. 2 as boxplots (A) and in Fig. 2 as a Bland-Altman plot (B).

#### Frequencies of Ki-67-positive immunohistochemistry using different cut-off values

Using 10, 14, 20 and 30 as cut-off values for Ki-67 staining, the frequencies obtained by using different cut-offs differed between Methods A and C, as shown in Table 1.

**Table 1:** Frequencies of Ki-67 using different cut offs.

Cut-offs	Method A (N=158)		Method C (N=158)	
	Nr. of pat. with Ki-67 ≤ cut-off	Nr. of pat. with Ki-67> cut-off	Nr. of pat. with Ki-67 ≤ cut-off	Nr. of pat. with Ki-67> cut-off
10	57 (36%)	101 (64%)	81 (51%)	77 (49%)
14	69 (44%)	89 (56%)	81 (51%)	77 (49%)
20	91 (56%)	67 (42%)	120 (76%)	38 (24%)
30	114 (72%)	44 (28%)	146 (92%)	12 (8%)

#### Summary statistics of OS and BCFI

The two clinical endpoints were OS and BCFI, which were collected from the Swiss patients in the IBCSG VIII and IX studies, and these values are presented in Table 2. These endpoints show 20 events in the subgroup with chemotherapy and 16 and 17 events, respectively, in the group without chemotherapy.

**Table 2:** Summary statistics of OS and BCFI.

Clinical endpoints	With Chemotherapy			Without Chemotherapy		
	N	Number of events	Median [95% CI]	N	Number of events	Median [95% CI]
OS (in years)	82	20	16.9 [16.2, NA]	76	16	16.8 [15.9, NA]
BCFI (in years)	82	20	Not reached	76	17	Not reached

Abbreviations: OS: overall survival, BCFI: breast cancer free interval.

#### Estimated OS and BCFI probabilities

**Figure 3:**
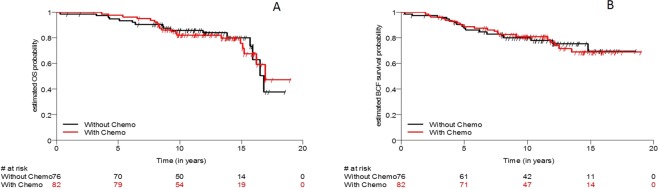
(**A)** Estimated overall survival (OS) probabilities. Patients in this cohort have similar outcomes in terms of OS. (**B**) Estimated breast cancer-free interval (BCFI) probabilities. Patients in this cohort have similar outcomes in terms of BCFI.

Patients in this Swiss collective enrolled in both treatment arms have similar outcomes as the entire IBCSG study population in terms of OS and BCFI. Estimated OS and BCFI probabilities are shown in Fig. 3(A,B).

#### Prognostic value of Ki-67 immunohistochemistry assessed by using Method A for determining OS/BCFI

Based on the estimated HR, a higher Ki-67 value did not result in significantly higher hazard ratio for OS and BCFI (all p values are greater than 0.05).

In Table 3A, we present the HR estimated using the univariate Cox regression model, which utilizes Ki-67 index assessed by using Method A as a numeric variable (the first row of the table) and a categorical variable based on different cut-off values (the second to fifth rows).

**Table 3:** Prognostic value of Ki-67 assessed by Method A (A) and by Method C (B).

(A) Variable Method A	OS		BCFI	
	HR (95% CI)	P value	HR (95% CI)	P value
Ki-67_A	1.01 (0.99–1.02)	0.54	1.01 (0.99–1.03)	0.20
Ki-67_A (>10 vs. <=10)	1.16 (0.58–2.33)	0.66	1.69 (0.82–3.50)	0.15
Ki-67_A (>14 vs. <=14)	1.20 (0.61–2.34)	0.59	1.54 (0.79–3.04)	0.20
Ki-67_A (>20 vs. <=20)	1.02 (0.53–1.98)	0.94	1.50 (0.79–2.86)	0.21
Ki-67_A (>30 vs. <=30)	1.02 (0.49–2.13)	0.94	1.16 (0.57–2.35)	0.68
**(B) Variable Method C**	**OS**		**BCFI**	
	**HR (95% CI)**	**P value**	**HR (95% CI)**	**P value**
Ki-67_C	1.00 (0.98–1.03)	0.87	1.01 (0.99–1.03)	0.47
Ki-67_C (>10 vs. <=10)	0.76 (0.39–1.48)	0.42	0.91 (0.48–1.74)	0.77
Ki-67_C (>14 vs. <=14)	0.76 (0.39–1.48)	0.42	0.91 (0.48–1.74)	0.77
Ki-67_C (>20 vs. <=20)	1.48 (0.71–3.09)	0.29	1.37 (0.66–2.84)	0.39
Ki-67_C (>30 vs. <=30)	0.99 (0.30–3.22)	0.98	1.23 (0.38–4.03)	0.73

Abbreviations: OS: overall survival, BCFI: breast cancer free interval.

#### Prognostic value of Ki-67 immunohistochemistry assessed by using Method C for determining OS/BCFI

In Table 3B, we present the HR estimated using the univariate Cox regression model with Ki-67 index assessed by using Method C as the numeric variable (the first row of the table) and a categorical variable based on different cut-off values (the second to fifth rows).

Notably, the HRs, 95% CIs and p values based on cut-off values of 10 and 14 are exactly the same due to the lack of a Ki-67 index when assessed by using Method C at cut-off values ranging from 10 to 15 (see Fig. 2(A)). Therefore, samples with Ki-67 levels equal or less than 14 are exactly the same as samples with Ki-67 levels equal or less than 10.

#### Predictive value of Ki-67 immunohistochemistry assessed by using Method A for determining OS

**Figure 4:**
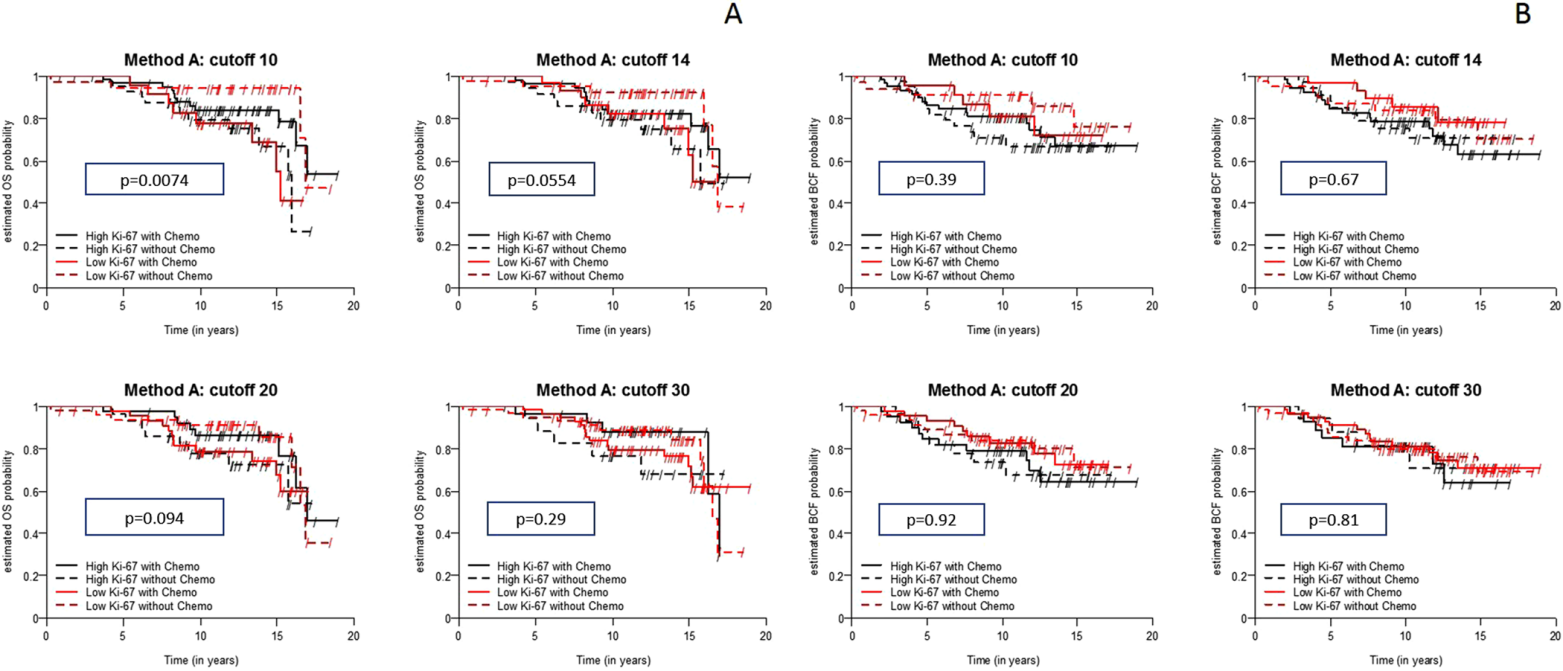
(**A)** Kaplan-Meier curves show the analyses of the overall survival (OS) of patients stratified by different cut-off values for Ki-67 levels, as measured using Method A and considering treatment allocation. (**B**) Kaplan-Meier curves show the analysis of the breast cancer-free interval (BCFI) in patients stratified by different cut-off values for Ki-67 levels measured using Method A and considering treatment allocation.

By using the cut-off of 10% and Method A, we found significant differences in the OS and predictive value at levels below and above this threshold (p=0.0074). The use of the cut-off of 14% almost reached statistical significance and showed only a trend towards an improved OS (p=0.0554). No other cut-off values produced significant differences. In Table 4(A), we separately presented the HRs and 95% CIs estimated using the univariate Cox regression model of OS with Ki-67 levels assessed by using Method A for the two treatment groups. The p value (*p value) presented in Table 4(A) was calculated for the interaction term based on the multivariate Cox regression model of OS with Ki-67, treatment group and their interaction. Notably, Tables 4(B) and 5 present the data in the same manner. In Fig. 4(A), the OS is stratified by different cut-off values for Ki-67 which were assessed by using Method A and considering treatment allocation. Based on the estimated HR presented in Table 4(A) a higher Ki-67 level (>10%) results in a lower hazard ratio (HR 0.48) for OS in patients treated with chemotherapy. For patients who were not treated with chemotherapy, the opposite effect was observed (HR 3.72). These data show that patients with Ki-67<=10% do not profit from chemotherapy and patients with Ki-67>10% might potentially have a benefit. Using 10% as the cut-off, this effect was statistically significant.

**Table 4:** Predictive value of Ki-67 assessed by Method A on OS (A) and on BCFI (B).

(A) Variables OS	HR (95% CI)		P value
	With Chemotherapy	Without Chemotherapy	
Ki-67_A	0.99 (0.95–1.02)	1.02 (1.00–1.04)	0.12
Ki-67_A (>10 vs. <=10)	0.48 (0.19–1.19)	3.72 (1.16–11.96)	**0.0074**
Ki-67_A (>14 vs. <=14)	0.67 (0.27–1.68)	2.64 (0.93–7.52)	0.0554
Ki-67_A (>20 vs. <=20)	0.64 (0.26–1.58)	2.02 (0.75–5.47)	0.094
Ki-67_A (>30 vs. <=30)	0.73 (0.27–2.03)	1.60 (0.55–4.62)	0.29
**(B) Variables BCFI**	**HR (95% CI)**		**P value**
	**With Chemotherapy**	**Without Chemotherapy**	
Ki-67_A	1.01 (0.98–1.04)	1.01 (0.99–1.03)	0.84
Ki-67_A (>10 vs. <=10)	1.23 (0.45–3.38)	2.31 (0.81–6.62)	0.39
Ki-67_A (>14 vs. <=14)	1.82 (0.66–5.01)	1.34 (0.52–3.50)	0.67
Ki-67_A (>20 vs. <=20)	1.45 (0.60–3.51)	1.55 (0.59–4.01)	0.92
Ki-67_A (>30 vs. <=30)	1.24 (0.50–3.13)	1.05 (0.34–3.22)	0.81

Abbreviations: OS: overall survival, BCFI: breast cancer free interval.

**Table 5:** Predictive value of Ki-67 assessed by Method C on OS (A) and BCFI (B).

(A) Variables OS	HR (95% CI)		P value
	With Chemotherapy	Without Chemotherapy	
Ki-67_C	0.98 (0.94–1.03)	1.01 (0.98–1.04)	0.34
Ki-67_C (>10 vs. <=10)	0.48 (0.20–1.19)	1.45 (0.53–3.99)	0.11
Ki-67_C (>14 vs. <=14)	0.48 (0.20–1.19)	1.45 (0.53–3.99)	0.11
Ki-67_C (>20 vs. <=20)	0.89 (0.29–2.71)	2.37 (0.86–6.54)	0.21
Ki-67_C (>30 vs. <=30)	0.98 (0.13–7.39)	0.97 (0.22–4.30)	0.96
**(B) Variables BCFI**	**HR (95% CI)**		**P value**
	**With Chemotherapy**	**Without Chemotherapy**	
Ki-67_C	1.01 (0.96–1.05)	1.01 (0.98–1.03)	0.88
Ki-67_C (>10 vs. <=10)	1.01 (0.42–2.44)	0.78 (0.29–2.12)	0.68
Ki-67_C (>14 vs. <=14)	1.01 (0.42–2.44)	0.78 (0.29–2.12)	0.68
Ki-67_C (>20 vs. <=20)	1.08 (0.39–2.97)	1.77 (0.62–5.03)	0.48
Ki-67_C (>30 vs. <=30)	1.58 (0.21–12.05)	1.11 (0.25–4.88)	0.83

Abbreviations: OS: overall survival, BCFI: breast cancer free interval.

#### Predictive value of Ki-67 immunohistochemistry assessed by using Method A for determining the BCFI

Ki-67 levels assessed by using Method A did not show a significant ability to predict BCFI in patients stratified by treatment allocation, as shown in Table 4(B) and Fig. 4(B).

#### Predictive value of Ki-67 immunohistochemistry assessed by using Method C for determining OS

**Figure 5:**
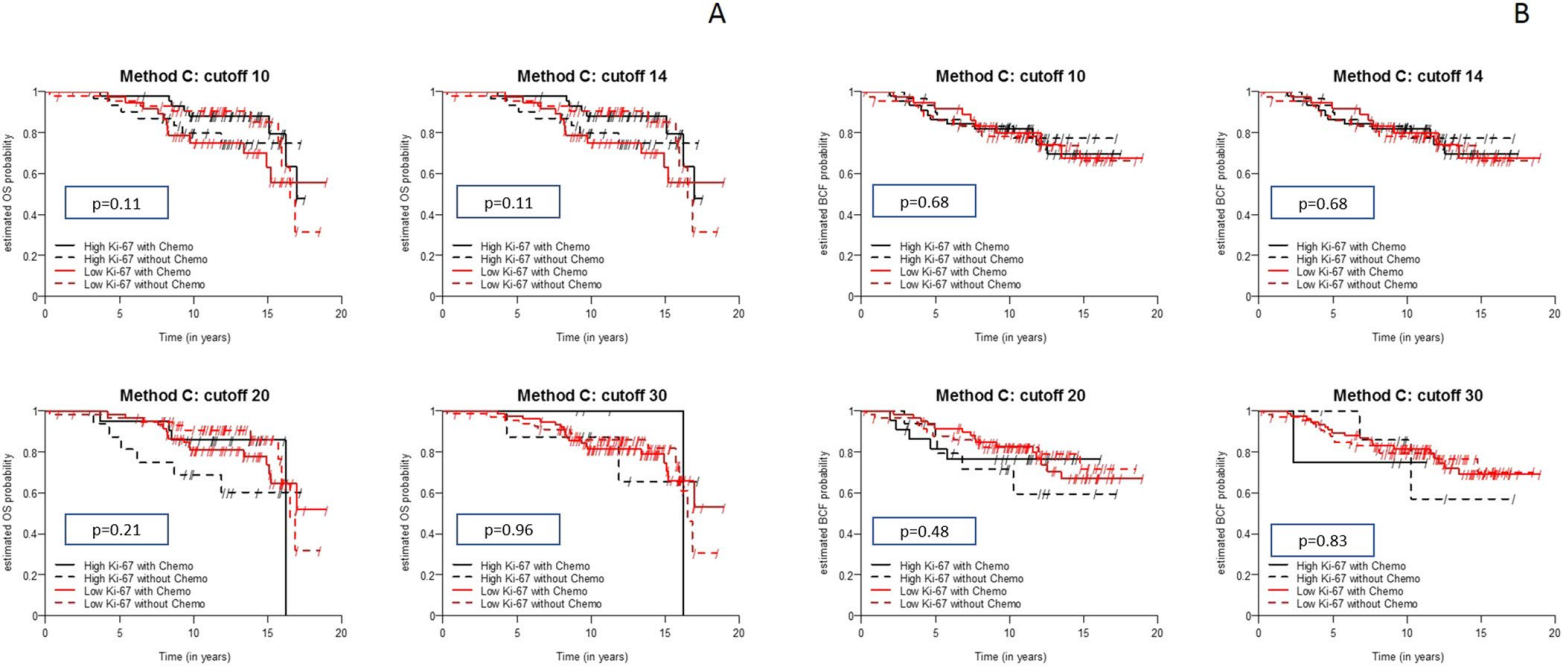
(**A**) Kaplan-Meier curves show differences in the overall survival (OS) of patients stratified by different cut-off values for Ki-67 levels measured using Method C and considering treatment allocation. (**B**) Kaplan-Meier curves show differences in the breast cancer-free interval (BCFI) in patients stratified by different cut-off values for Ki-67 levels measured using Method C and considering treatment allocation.

Ki-67 levels assessed by using Method C did not display a significant ability to predict OS in patients stratified by treatment allocation, as shown in Table 5(A) and Fig. 5(A). However, there was a similar tendency for Method C compared to Method A with respect to OS.

#### Predictive value of Ki-67 immunohistochemistry assessed by using Method C for determining BCFI

Ki-67 levels did not display a significant ability to predict BCFI in patients stratified by treatment allocation, as shown in Table 5(B) and Fig. 5(B).

#### Correlation between Ki-67 immunohistochemistry and the mRNA-dependent luminal subtype assessment

**Figure 6:**
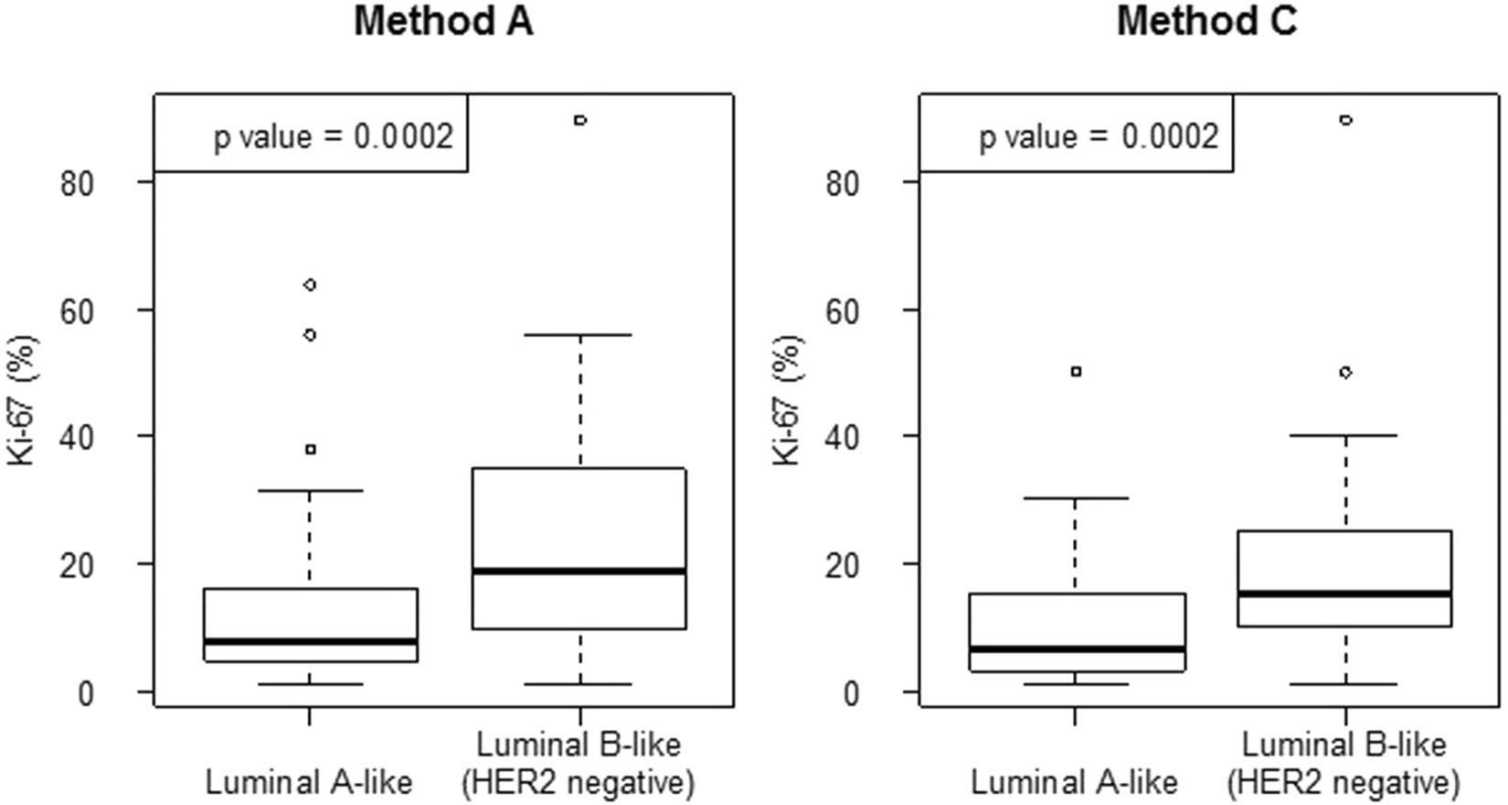
A significant correlation was observed between the immunohistochemical Ki-67 assessments (methods A and C) and classification with the Ki-67 mRNA-dependent *Luminal A-* and *Luminal B-like* intrinsic subtypes using MammaTyper.

A significant correlation was observed between the immunohistochemical assessments (methods A and C) and the classification of the intrinsic subtypes as *Luminal A-like* or *Luminal B-like* with the MammaTyper kit: Range for Ki-67 values in both Method A and C was 1.00 to 90.00. As shown in Fig. 6, patients with MammaTyper *Luminal B-like* tumours generally presented higher Ki-67 values in IHC than patients with the *Luminal A-like* subtype.

#### Correlation between Ki-67 mRNA-dependent *Luminal A-* and *Luminal* B-like subtypes and OS/BCFI

**Figure 7:**
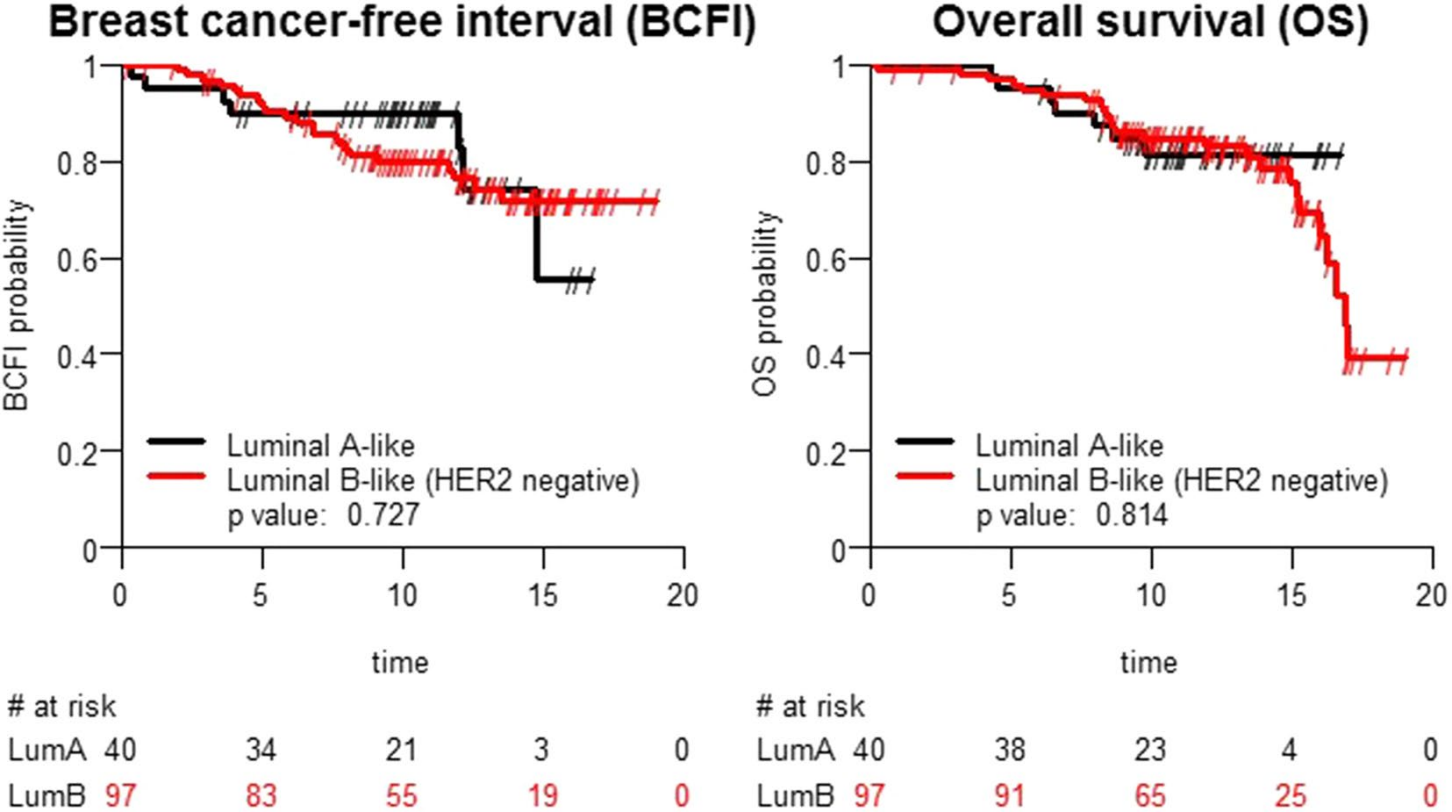
Correlation between overall survival (OS), breast cancer-free interval (BCFI) and the Ki-67 mRNA-dependent *Luminal A-* and *Luminal B-like* intrinsic subtype assessment with MammaTyper (the results were not statistically significant).

In this cohort, we did not identify any significant correlations between OS/BCFI and the Ki-67 mRNA assessment, as shown in Fig. 7. Notably, the MammaTyper Ki-67 mRNA cut-off corresponds to a 20% IHC cut-off, which was not significant in this cohort, as shown above and determined by using IHC.

## Discussion

In the SAKK 28/12 validation phase, we analysed the prognostic and predictive value of Ki-67 immunohistochemistry assessed by using the most reproducible methods selected from the pilot phase of SAKK 28/12^27^. The old, archived paraffin blocks containing breast cancer tissues from patients in the IBCSG VIII and IX clinical trials and treated at the time of the trials in Switzerland were used in this study, and the corresponding clinical outcomes were also used for this project^27,30,31^. The median follow-up from randomization in Trial VIII was 12 years and in Trial IX was 13 years^33,34^. Additionally, we also assessed Ki-67 mRNA levels and analysed the association with clinical outcomes, as we reported in an earlier paper, and Ki-67 mRNA levels are found to be highly reproducible^32^.

As shown in the present study, a Ki-67 index obtained using immunohistochemical method A (exact counting of tumour cells at the tumour periphery) with a cut-off of 10% predicts the OS of patients treated with chemotherapy. Although other results regarding the predictive and prognostic values were not statistically significant, Ki-67 levels measured by using Method A represent a potential prognostic factor for BCFI based on the estimated HRs and confidence intervals, which indicated a higher risk of recurrence in patients with higher Ki-67 levels.

The identification of the optimal method or methodologies for the assessment of proliferative activity in breast cancer has been the subject of several previous studies in the last decade since the introduction of Ki-67 as a routinely assessed parameter in hormone receptor-positive breast cancer specimens^1–12^. These studies analysed different types of visual assessments and digital analyses to test whether one method outperforms the other or if these methods yield the same results in terms of reproducibility^1–6,8,9,11–14^. Based on the currently available published data, a trend that both visual and digital analyses result in a similar inter-rater coefficient has been observed, enabling the diagnostic use of both approaches^9,15–19^. However, the optimal methodology for assessing Ki-67 levels in breast cancer that fulfils the criteria of perfect inter-rater and inter-laboratory reproducibility has not yet been identified^4,6,8,10,13,19,23,27^. In contrast to midrange proliferative cancers where reproducibility remains an issue, the inter-rater reliability is considerably better for low and high proliferative cancers^4,6,8,10,11,13,19,23,27^. Intra-tumour heterogeneity and the area chosen for the Ki-67 assessment appear to be the most crucial factors, in addition to pre-analytical inter-laboratory differences at the current time^4–6,8,10,13,19,23,27^. This heterogeneity remains a relevant factor for Ki-67 and gene-signature tests.

The first descriptions of utilizing cut-off values with Ki-67 levels to make clinical decisions and to estimate prognosis were derived from Ki-67 measurements obtained from tumour samples in the IBCSG VIII and IX prospective clinical trials^1,2,7,12,14,17,35^.

One of the first sources of data on Ki-67 power measured in the IBCSG VIII and IX trials showed that the Ki-67 labelling index does not predict a benefit from adding chemotherapy to endocrine therapy but, rather, indicates a worse disease-free survival rate regardless of the treatment modalities and, thus, provides important prognostic information^35^.

The median Ki-67 values, as assessed by central pathology, for tumours in IBCSG VIII and IX were 19%^35^.

Since the original description in 2008, which stated that a cut-off of 14% for the Ki-67 level differentiates between Luminal A and Luminal B tumours, the cut-off value has periodically undergone adjustments, such as shifting from 20% to 30% or being described as simply low and high, depending on the midrange Ki-67 levels measured at a specific pathology institution, and these modifications are still underway even currently^1,2,4,6,8,11,13,19,23,27^. This change is probably one reason why our study used a lower optimal and significant Ki-67 cut-off value (10%) to assess overall survival, although the median Ki-67 level was 17.7% for method A. Further explanations for our discrepancy from the original definition of 14% are most likely the smaller sample size and the observation of fewer events in both arms of the Swiss subset of the IBCSG VIII and IX cohorts, although applying a 14% cut-off value with Method A almost reached statistical significance in this subset.

The difficulties in defining the optimal cut-off value and the most reproducible Ki-67 assessment methods has led to adjustments in the clinical guidelines as well, as the current recommendations of the St. Gallen 2017 Consensus Conference do not include cut-offs but instead state that low and high Ki-67 categories, in accordance with the midrange Ki-67 values of the specific pathology laboratory, should be applied^1,2,8,10,13,19,23,27^. Nevertheless, Ki-67 levels greater than 20–25, regardless of the assessment methodology, are probably the best approximate cut-off values to estimate risk of death compared to lower values and to decide whether additional adjuvant chemotherapy should be administered^2,12^.

Alternative methods to immunohistochemical Ki-67 assessments, such as mRNA-based analyses, were recommended in recent studies, as inter-laboratory reproducibility with ICC values of 0.980–0.998 revealed the excellent agreement of quantitative measurements for Ki-67 levels measured using MammaTyper^13,32^. Another recent mRNA-based study assessing Ki-67 levels with STRAT4 showed a good correlation with Ki-67 immunohistochemistry at a 30% cut-off^36^. However, the clinical utility, particularly in the intermediate range, has not yet been confirmed.

In the present study, Ki-67 IHC assessed by using methods A and C correlated well with the mRNA-based assessments of *Luminal A-like* and *Luminal B-like* (HER2 negative) tumours, although Methods A and C produced different Ki-67 values, namely, mean values of 21.3 and 16.1, respectively. This observation has been reported in recent studies, showing that different assessment methodologies using both visual and digital measurements result in different mean and median Ki-67 levels^4–6,23,28,37,38^. The lack of any significant correlations between the mRNA-based Ki-67-dependent *Luminal A-like* and *Luminal B-like* subtype assessment and OS/BCFI in our study is probably due to the smaller sample size in the Swiss cohort and the focus on grade 2 tumours, which is in contrast to the entirety of the IBCSG VIII and IX clinical trials. Within this restricted cohort, only the 10% Ki-67 IHC cut-off reached significance, while the MammaTyper MKI67 cut-off correlated with a 20% Ki-67 cut-off. Furthermore, intra-tumour heterogeneity and the analysis of different tumour areas in different tumour blocks from the same tumour of each patient differed from the analyses applied in the original IBCGS VIII and IX subsets and should be considered when interpreting divergent results.

The question of the optimal tissue, such as biopsy, surgical specimen or tissue-micro-arrays (TMA), to assess the Ki-67 index in breast cancer is controversial and has been addressed in the literature^9,10,25,26,37–39^. As shown in several previous studies, Ki-67 levels obtained from the same tumour, whether obtained via TMA, core biopsy or surgical specimen, differ due to intra-tumour heterogeneity, which must be considered in clinical practice if different tissue specimens are available^37–39^. In our study, we restricted the analysis to surgical specimens, which was similar to the original IBCSG VIII and IX cohorts. Nevertheless, in daily routine pathological diagnostics, core biopsies are increasingly considered the primary source for Ki-67 assessments regarding both adjuvant therapy decisions and preoperative chemotherapy selection^3,8,10,16,17^. As described above, optimal Ki-67 cut-off values in core biopsies for predicting the response to preoperative chemotherapy range from 15–30%^3,16,17,40^.

## Conclusions

In summary, different Ki-67 assessment methodologies affect the correlations with overall survival and the breast cancer-free interval in patients with moderately differentiated breast cancer. Based on our results, method A (counting cells using visual assessment) using a cut-off of 10% was a predictive marker of OS in patients who were not treated with chemotherapy. Higher Ki-67 index was not associated with outcome and using the 10% Ki-67 cut-off there was an opposite association for patients with and without chemotherapy.

The results in this study are hypothesis generating and additional validation of these finding appears warranted. The issue of Ki-67 assessment in breast cancer in terms of the methodology and optimal cut-off values, particularly in midrange samples, remains a challenge, and further studies analysing correlations and prospective clinical trials are needed.

## Data Availability

All data and study materials are available upon request without any restrictions.
